# Multi-omics analysis reveals metabolic disruptions in cardiac tissues of aging nonhuman primates with spontaneous type 2 diabetes

**DOI:** 10.18632/aging.206261

**Published:** 2025-06-02

**Authors:** Yaowen Liu, Jingning Yu, Hao Hu, Shaoxia Pu, Haiyan Wu, Yunyu Ma, Wenhui Yang, Chongye Fang, Fei Sun, Haizhen Wang

**Affiliations:** 1 College of Veterinary Medicine, Yunnan Agricultural University, Kunming 650021, China; 2 College of Food Science and Technology, Yunnan Agricultural University, Kunming 650021, China; 3 Department of Cardiology, The Affiliated Hospital of Kunming University of Science and Technology, The First People's Hospital of Yunnan Province, Kunming 650032, China; 4 Department of Cardiology, Fuwai Yunnan Cardiovascular Hospital, Kunming 650102, China

**Keywords:** aging, type 2 diabetes, nonhuman primates, cardiology, liver-heart crosstalk

## Abstract

Background: The complex interplay between type 2 diabetes (TM) and obesity, particularly in aging populations, is increasingly recognized for its significant contribution to cardiac dysfunction. However, the metabolic changes within cardiac tissues that underlie this relationship remain poorly understood.

Methods: We employed a multi-omics approach to investigate the metabolic alterations in cardiac tissues of aging nonhuman primates with spontaneous TM and obesity. Comprehensive analysis was conducted on left ventricular heart tissues from control (CON), obesity (OB), and TM groups, each comprising three aging monkeys. Proteomic data were analyzed using label-free mass spectrometry, and lipidomic profiles were determined using targeted metabolomic assays.

Results: Our analysis uncovered significant metabolic perturbations in both the OB and TM groups relative to controls. Notably, the TM group showed alterations in cardiac metal ion metabolic proteins and a disruption in the liver-heart crosstalk, suggesting a derailment in the heart's metabolic support system. This was further exacerbated by reduced levels of short-chain acylcarnitines and lysophosphatidylcholines (lysoPCs), coupled with an increase in C18:2 acylcarnitines. A progressive decline in amino acid levels was observed from the control to OB to TM groups, indicating a stepwise deterioration in cardiac metabolic remodeling.

Conclusions: This multi-omics study in aging nonhuman primates provides novel insights into the metabolic dysregulations associated with TM and obesity in cardiac tissues. The observed metabolic changes highlight potential therapeutic targets for prevention or mitigating the cardiac complications of TM.

## INTRODUCTION

The global population, including China, is experiencing rapid aging due to declining fertility rates and increasing longevity. By 2019, China's elderly population—those aged 65 and above—surpassed 176 million, comprising 13% of the total population [1]. This aging process is occurring more swiftly in China than in other low- and middle-income countries [2]. Concurrently, the risk of heart failure rises significantly with age, becoming particularly pronounced among individuals over the age of 85. This demographic shift is exerting an increasing strain on healthcare systems worldwide.

A key driver of this trend is the cardiometabolic remodeling that accompanies aging. This remodeling, often a result of systemic metabolic changes, initiates a cascade of effects that can lead to structural and electrical alterations within the heart, culminating in cardiac dysfunction [3]. For example, an increased reliance on glucose metabolism can lead to the accumulation of aspartate in cardiomyocytes, a condition associated with ventricular hypertrophy in hypertrophic cardiomyopathy [4]. These changes underscore the need for a deeper understanding of the aging process and its impact on cardiometabolic health.

Aging significantly alters the whole body metabolism. First, insulin sensitivity declines [5–7]. Meanwhile, hepatic glucose output is similar in young and old [8], leading to a gradual decline in blood sugar regulation. Second, lipid metabolism also changes with age. Data show that obesity prevalence is notably higher in older adults compared to younger populations [9, 10], with significant differences in adipose tissue quality and distribution [11]. Increased adipose mass and visceral fat accumulation are characteristic of age-related ectopic fat distribution [12], which negatively impacts metabolic health and elevates the risk of conditions such as type 2 diabetes (TM) [13], which increase and decrease fatty acids and glucose utilization in the heart respectively.

Globally, the impact of obesity and TM on heart failure is profound, particularly among aging populations, and it induces cardiometabolic remodeling similar to that caused by aging. Patients with TM face twice the risk of heart failure compared to non-diabetic individuals, and TM is an independent factor associated with higher mortality and readmission rates among heart failure patients [14–17]. From 1990 to 2017, China experienced a 29.9% increase in heart failure cases, paralleling the rise in obesity and TM incidence [18]. In the United States, 44% of hospitalized heart failure patients have either type 1 or type 2 diabetes [16]. These findings highlight the critical link between obesity, TM and heart failure, underscoring the need for targeted interventions.

However, the precise characterization of the cardiometabolic shifts in the aging population with obesity or TM remains an area that requires in-depth study. While existing research has established a link between TM and an increased risk of heart failure, the underlying mechanisms driving these changes, especially in the context of aging, are not fully understood. This knowledge gap is critical, as a deeper understanding of the molecular and physiological processes could lead to the development of more effective preventative and therapeutic strategies.

To address these critical questions, we conducted comprehensive large-scale screening for spontaneous obesity or TM in an aging macaque population. Subsequently, we conducted a comparative analysis of the proteomic, metabolomic, and lipidomic profiles in the left ventricle of selected monkeys. This comprehensive approach allows us to elucidate a global picture of the metabolic changes that occur in aging monkeys as they transition from obesity to TM.

## RESULTS

### Alteration of proteomics profile in the OB and TM group compared to the CON

**Figure 1:**
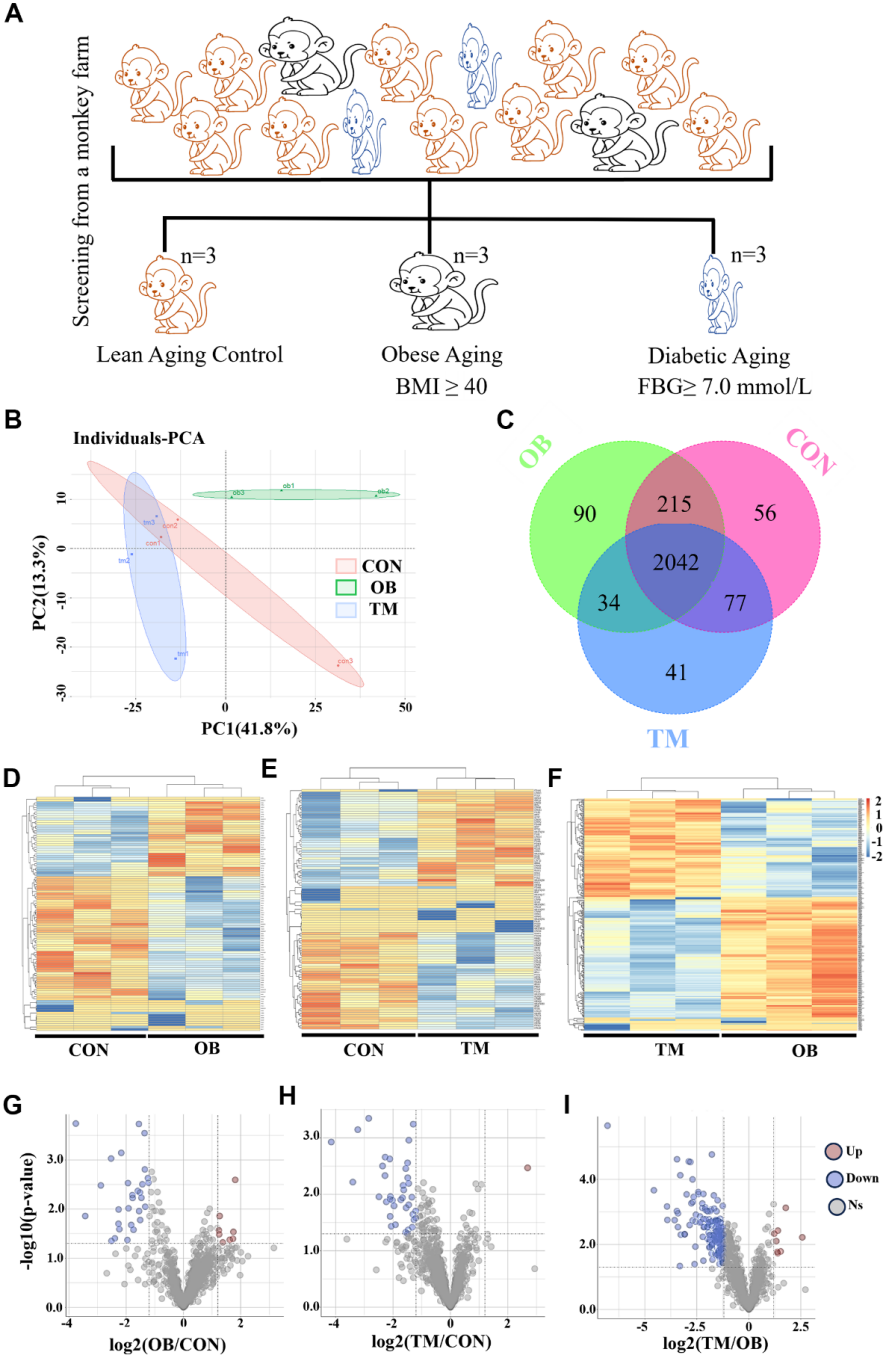
**Proteomic analysis reveals cardiac tissue alterations in obesity (OB) and type 2 diabetes (TM) monkeys.** (**A**) Identification and screening process for spontaneously obese and type 2 diabetic aging monkeys from a monkey farm. (**B**) PCA plot demonstrating the distinct proteomic profiles among the CON, OB, and TM groups. (**C**) Venn diagram illustrates the overlap and uniqueness of detected proteins across the three groups. (**D**–**F**) Heatmaps depicting the expression patterns of differentially expressed proteins in each group. (**G**–**I**) Volcano plots highlighting the significant differentially expressed proteins selected for further analysis.

To explore the metabolic consequences of aging and TM on cardiac function, we conducted a comprehensive proteomic analysis. We screened a large cohort of aging monkeys from a monkey farm and identified three groups: aging with obesity (OB), aging with TM, and healthy aging controls (CON) (Figure 1A). Body weight, body mass index, and fasting blood glucose data for these groups are detailed in Supplementary Table 4. Through label-free mass spectrometry, we profiled the left ventricular proteomes of these groups and performed a principal component analysis (PCA), which revealed significant proteomic differences (Figure 1B).

A total of 2555 proteins were detected across all groups (Supplementary Table 1), with 90 and 41 proteins uniquely present in the OB and TM groups, respectively, and 56 proteins exclusive to the CON group (Figure 1C). The heatmaps in Figure 1D to F present the comprehensive differential protein profiles of the three groups. Proteins with more than a 1.2-fold change in expression and a p-value less than 0.05 were considered as candidates for further analysis, providing a robust dataset to investigate the molecular signatures of cardiac remodeling in the context of aging and OB or TM.

Comparative analysis revealed that, relative to the CON group, 8 and 29 proteins were upregulated and downregulated in the OB group, respectively, while 1 and 38 proteins were upregulated and downregulated in the TM group. When the TM group was compared to the OB group, 8 and 181 proteins were found to be upregulated and downregulated, respectively (Figure 1G–1I).

### Multiple extracellular matrix (ECM) related proteins were affected by obesity or diabetes

**Figure 2:**
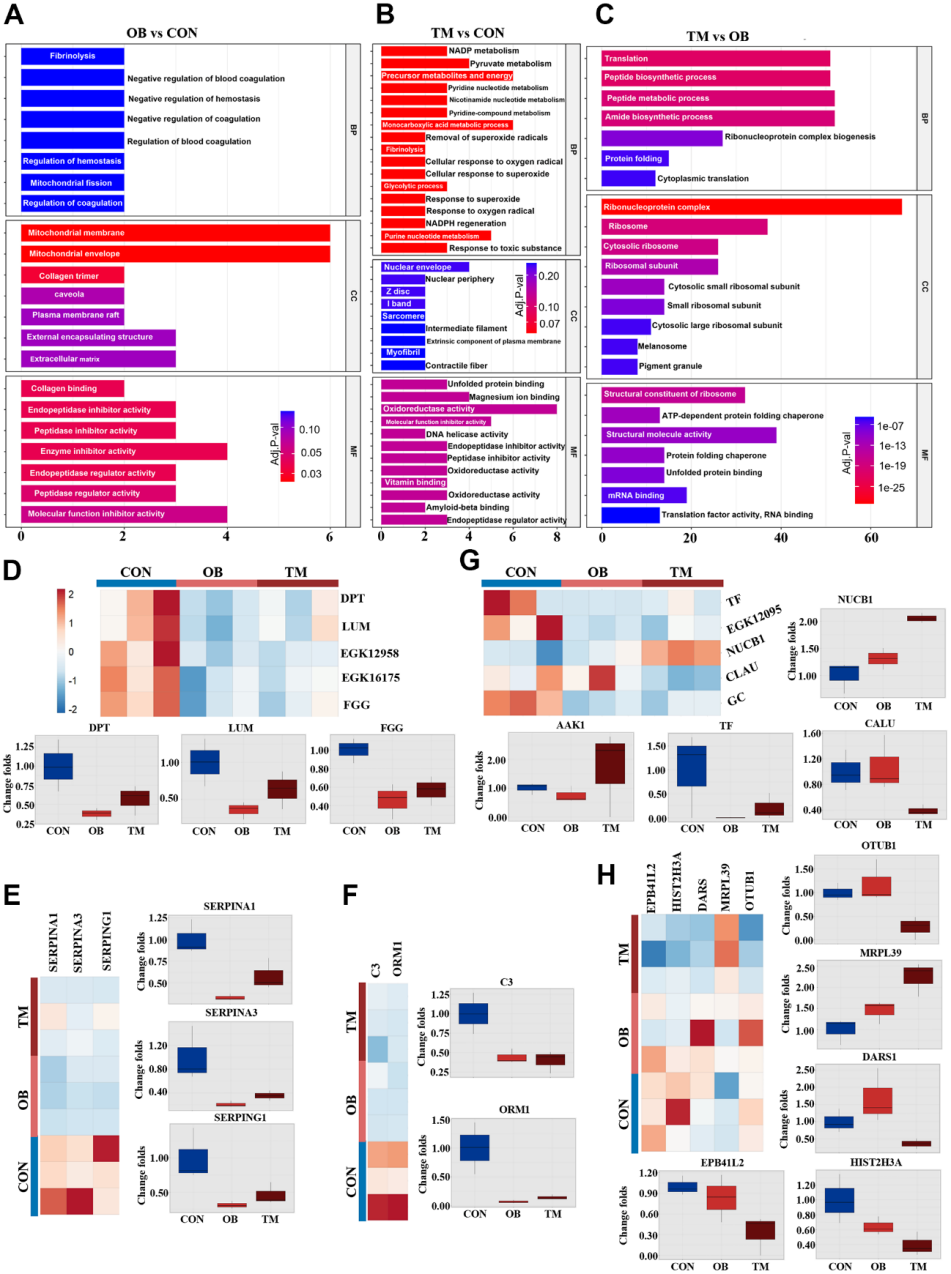
**Comparative analysis of cardiac tissue pathways in the OB and TM groups.** (**A**–**C**) Display the Gene Ontology (GO) analysis comparing the CON, OB, and TM groups in a pairwise manner, highlighting the most affected pathways. (**D**) Presents the differentially expressed typical extracellular matrix (ECM) related proteins across the CON, OB, and TM groups. (**E**) Illustrates the differential expression of serpin family members, which play a role in the regulation of the complement cascade. (**F**) Depicts the differentially expressed immune-related proteins in cardiac tissue among the CON, OB, and TM groups. (**G**) Shows the differentially expressed proteins related to metal ion metabolism in cardiac tissue across the CON, OB, and TM groups. (**H**) Profiles other differentially expressed proteins in cardiac tissue that were identified in the CON, OB, and TM groups.

To explore the effects of differential protein expression on cardiac function, we undertook GO and KEGG analyses of our proteomic dataset. GO analysis revealed significant alterations in extracellular matrix and mitochondrial component pathways in the obesity (OB) group. In contrast, the triglyceride metabolism (TM) group exhibited broader disruptions in energy metabolism and myocardial structural pathways compared to the healthy aging control (CON) group (Figure 2A, 2B). Additionally, the TM group displayed unique changes in ribosome complex, nucleic acid binding, and peptide biosynthetic pathways relative to the OB group (Figure 2C).

Building on these findings, KEGG analysis pinpointed the complement and coagulation cascade, along with cytoskeletal pathways, as being most affected in the OB group. In contrast, the TM group showed more pronounced disruptions in focal adhesion and muscle cytoskeletal pathways (Supplementary Figure 1).

Our proteomic analysis further revealed significant downregulation of key extracellular matrix proteins Dermatopontin (DPT), Lumican (LUM), and Haptoglobin (EGK_12958) in both OB and TM groups relative to controls. This was accompanied by substantial downregulation of Fibrinogen gamma chain protein (FGG) and beta chain protein (EGK_16175), indicating a potential disruption in the coagulation cascade (Figure 2D).

The serpin family members SERPINA1 and SERPINA3 were significantly downregulated in both OB and TM groups, consistent with alterations in the complement system. Another serpin family member, SERPING1, which regulates the complement cascade, was also downregulated, along with a decrease in the major complement component C3 and the immune-related protein Alpha-1-acid glycoprotein (ORM1) (Figure 2E, 2F). This downregulation across OB and TM groups points to a common impact on the immune and complement systems.

Proteins associated with metal ion metabolism were also affected, with Nucleobindin-1 (NUCB1) increased and Calumenin (CALU) decreased in the TM group. Vitamin D binding protein, GC, showed a consistent decrease in both groups. Additionally, iron metabolic regulation was altered, as Ap2 associate kinase (AAK-1) was elevated in the TM group, while transferrin (TF) and serotransferrin (EGK_12095) were decreased in both groups (Figure 2G).

Lastly, protein homeostasis factors, including Histone H3 (HIST2H3A), Aspartyl-tRNA synthetase (DARS1), 39S ribosomal protein L39 (MRPL39), and Ubiquitin thioesterase (OTUB1), exhibited differential regulation between the OB and TM groups (Figure 2H).

To validate the proteomic findings, we assessed the expression of key proteins—TF, ORM1, C3, NUCB1, and serpin family A member 1 (SERPINA1)—via Western blot analysis. The results corroborated the proteomic data, exhibiting consistent trends across the OB, TM, and CON groups (Supplementary Figure 2). These findings confirm the reliability and accuracy of the label-free mass spectrometry approach, reinforcing the observed metabolic and structural perturbations in the DCM myocardium.

### Metabolic profiling reveals distinct cardiac metabolite alterations in obesity and type 2 diabetes

**Figure 3:**
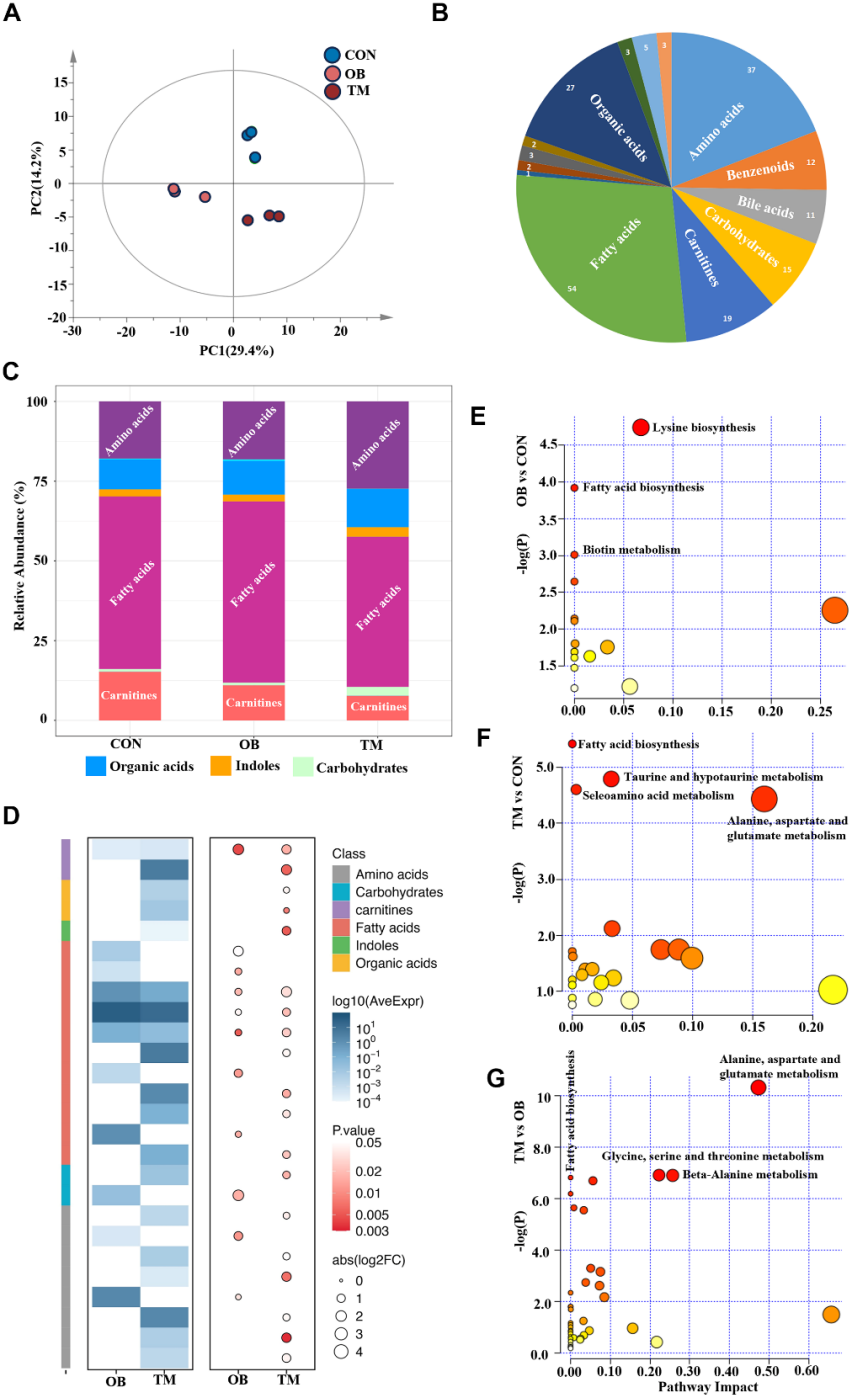
**Metabolic profiling reveals distinct metabolite patterns in the CON, OB, and TM groups.** (**A**) PCA plot elucidates the separation of metabolomic profiles among the CON, OB, and TM groups, indicating significant differences. (**B**) Categorization of detected metabolites from the comprehensive metabolomics study, showcasing the diversity of metabolites analyzed. (**C**) Comparative analysis of metabolite clusters' relative abundance in the CON, OB, and TM groups, highlighting variations in metabolite distribution. (**D**) Heatmap illustrating the differential metabolite profiles across the CON, OB, and TM groups. The color intensity represents the logarithm of average expression levels (log10(AveExpr)) for different compounds across two conditions (OB and TM) compared with CON. The size of the circles corresponds to the absolute value of the logarithm of the fold change (abs(log2FC)), and the color of the circles represents the p-value, indicating the statistical significance of the differences observed. The compounds are categorized by class, as indicated by the color key on the right side of the image. (**E**–**G**) Pairwise plots of Metabolic Pathways Enrichment Analysis (MPEA) for the CON, OB, and TM groups, detailing the enrichment of specific metabolic pathways within each group.

To determine whether altered protein expression corresponds to changes in myocardial metabolites, we conducted a comprehensive metabolomic analysis on left ventricular heart tissue from the three study groups (Supplementary Table 2). This analysis was designed to explore the downstream metabolic effects of the protein expression changes observed in our proteomic study. PCA revealed significant metabolic distinctions among the groups, highlighting the divergent metabolite compositions (Figure 3A). In total, we identified 194 measurable and reproducible metabolites, encompassing 54 fatty acids, 37 amino acids, 27 organic acids, and 19 carnitines (Figure 3B).

The metabolic profile of the TM group notably deviated from that of the CON and OB groups, exhibiting a reduction in fatty acids and carnitine levels alongside an increase in amino acids and carbohydrate content (Figure 3C). These observations suggest that the metabolic shifts in the TM group may be indicative of an adaptive response or a pathological change in energy metabolism. Heatmaps visually depicted the profiles of differential metabolites, providing a clear overview of metabolic variations (Figure 3D).

To determine which pathways were affected by obesity or diabetes, the Metabolic Pathways Enrichment Analysis (MPEA) was applied. MEPA revealed that lysine biosynthesis, fatty acid biosynthesis, and biotin metabolism were the most significantly affected pathways in the OB group (Figure 3E). In contrast, the TM group showed pronounced disruptions in amino acids metabolism, taurine metabolism, and fatty acid biosynthesis when compared to the CON group (Figure 3F). A comparative analysis from OB to TM revealed that amino acids metabolism and fatty acid biosynthesis were the primary metabolic pathways that changed, indicating a shift in metabolic flux associated with the progression from obesity to TM (Figure 3G).

### Comprehensive metabolomic profiling unveils diverse metabolic disruptions in obesity and type 2 diabetes

**Figure 4:**
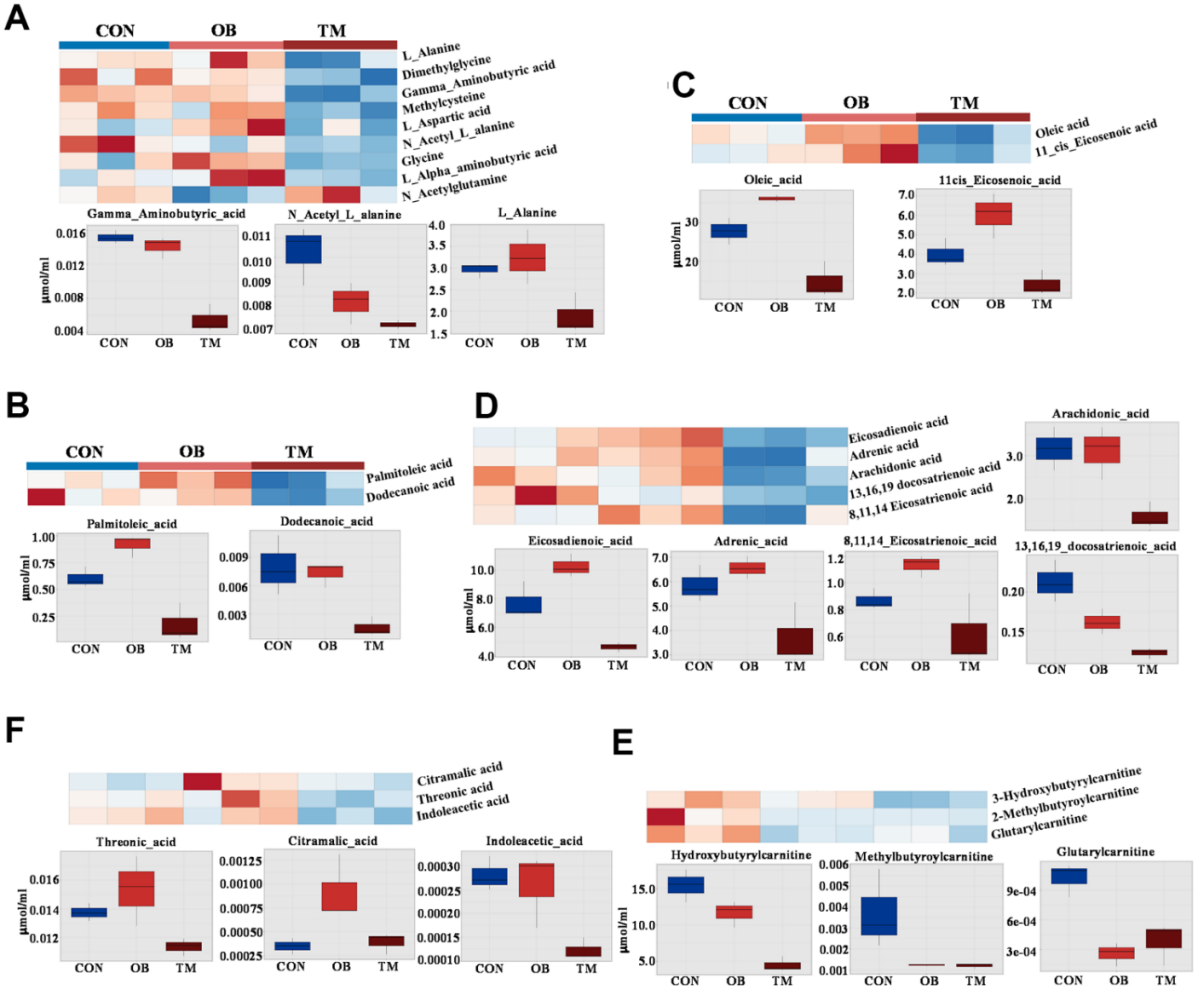
**Comparative metabolite profiles in control (CON), obesity (OB), and type 2 diabetes (TM) groups.** (**A**) Heatmap and box graph depicting the differential amino acid levels in the CON, OB, and TM groups, showcasing distinct metabolic patterns. (**B**) Heatmap and box graph illustrating the variations in saturated fatty acids in the CON, OB, and TM groups, highlighting metabolic alterations. (**C**) Heatmap and box graph displaying the differences in monounsaturated fatty acids across the CON, OB, and TM groups. (**D**) Heatmap and box graph showing the changes in polyunsaturated fatty acids among the CON, OB, and TM groups. (**E**) Heatmap and box graph representing the differential short-chain carnitine levels in the CON, OB, and TM groups, indicating shifts in fatty acid metabolism. (**F**) Heatmap and box graph presenting the differential organic acid levels in the CON, OB, and TM groups, reflecting alterations in key metabolic pathways.

In our metabolomic analysis, we utilized univariate statistical analysis to discern 24 metabolites that were significantly differentially expressed, providing insights into the metabolic repercussions of obesity and TM within an aging context. The metabolism of amino acids was particularly perturbed; L-Alanine was significantly downregulated by 36% in the TM group relative to the control group, with no significant change observed in the obesity group. This pattern was echoed by N-Acetyl-L-alanine, another alanine derivative, which also saw a 36% decrease in the TM group. Additionally, Gamma-Aminobutyric-acid (GABA) was specifically and significantly downregulated in the TM group, with a pronounced 63% reduction. Other amino acids including L-Alpha-aminobutyric acid, Dimethylglycine, Glycine, and Methylcysteine were significantly downregulated in TM groups, albeit from a low baseline. In contrast, N-Acetylglutamine, and L-Aspartic acid were selectively affected by obesity, indicating distinct metabolic sensitivities to these conditions (Figure 4A).

The metabolism of fatty acids was predominantly affected in the TM group. Palmitoleic acid, a saturated fatty acid, showed a paradoxical 25% increase in the OB group but a substantial 50% decrease in the TM group when compared to controls. This dichotomy was also observed in Dodecanoic acid, with the latter decreasing by 47% in the TM group. The mono-unsaturated fatty acids, oleic acid and 11-cis-Eicosenoic acid, exhibited a non-significant trend towards increase in the obesity group but a significant 47% and 38% decrease in the TM group respectively. This pattern was mirrored by poly-unsaturated fatty acids such as eicosadienoic acid, adrenic acid, and 8,11,14-Eicosatrienoic acid, which showed a mild increase in the obesity group followed by a sharp decrease in the TM group. The long-chain fatty acids, 13,16,19-docosatrienoic acid, demonstrated a stepwise reduction from both obesity to TM groups, with the TM group exhibiting the most significant decreases. Arachidonic acid significantly downregulated in the TM group (Figure 4B–4D).

In concordance with the observed decline in fatty acid content in the TM group, short-chain carnitines were significantly downregulated. Hydroxybutyrylcarnitine and methylbutyroylcarnitine were the primary contributors to this decrease, with substantial reductions observed in both the obesity and TM groups compared to controls. Even the low-abundance Glutarylcarnitine was significantly downregulated in both groups, indicating a broad impact on fatty acid metabolism (Figure 4E).

Furthermore, threonic acid, a sugar acid, was significantly downregulated by 17.4% in the TM group. Other organic acids including Citramalic acid and Indoleacetic acid also showed significant changes, albeit from very low baseline levels, suggesting a complex metabolic reprogramming in the TM group (Figure 4F).

### Lipidomic profiling reveals distinct lipid alterations in obesity and type 2 diabetes

**Figure 5:**
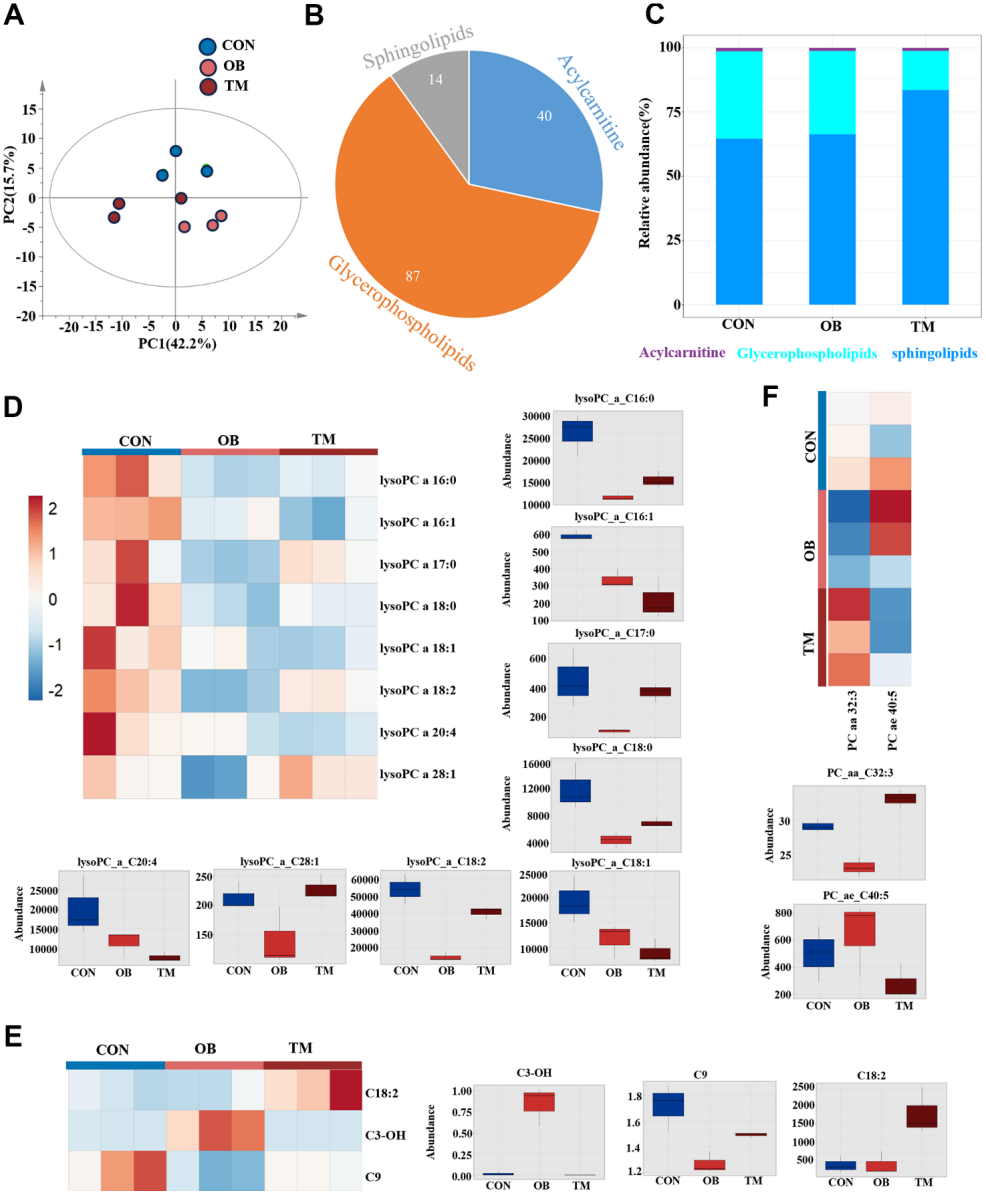
**Lipidomic variations among CON, OB, and TM groups.** (**A**) PCA plot delineating the lipidomic distinctions among the CON, OB, and TM groups. (**B**) Pie graph illustrates the ratio of detected sphingolipids, acylcarnitines, and glycerophospholipids across the CON, OB, and TM groups. (**C**) Bar graph shows the relative abundance of sphingolipids, acylcarnitines, and glycerophospholipids in the CON, OB, and TM groups. (**D**) Heatmap and box graph displaying lysoPC levels in the CON, OB, and TM groups, revealing metabolic variations. (**E**) Heatmap and box graph presenting acylcarnitine levels in the CON, OB, and TM groups, indicating alterations in lipid metabolism. (**F**) Heatmap and box graph depicting phosphatidylcholine (PC) levels in the CON, OB, and TM groups, highlighting differences in lipid profiles.

Following our metabolomic findings that indicated suppressed short-chain acylcarnitines in both OB and TM groups, we conducted a lipidomic study to further elucidate the lipid changes (Supplementary Table 3). We measured acylcarnitines, glycerophospholipids, and sphingolipids and observed significant differences among the CON, OB, and TM groups using PCA (Figure 5A). A total of 87 glycerophospholipids, 40 acylcarnitines, and 14 sphingolipids were detected (Figure 5B). Notably, the TM group exhibited lower levels of glycerophospholipids and higher levels of sphingolipids compared to the CON and OB groups (Figure 5C).

Through pairwise T-tests and one-dimensional statistical analysis, we identified 13 significantly differential lipid species, including 3 acylcarnitines, 8 lysoPCs, and 2 PCs. (Figure 5D–5F). In both the OB and TM groups, we observed a decrease in lysoPC species C16:0, C17:0, C18:0, C18:2, and C28:1, with the most substantial reductions in the OB group. Notably, lysoPC C16:0 levels were significantly decreased by 66% and 48% in the OB and TM groups, respectively, relative to controls. The levels of lysoPC C18:0 were also reduced by 62% and 41% in the OB and TM groups, respectively, though the change in the TM group was not significant compared to controls. LysoPC species C16:1, C18:1, and C20:4 showed a stepwise downregulation from the control group to the OB and TM groups, suggesting a progressive impact of obesity and diabetes on lipid metabolism (Figure 5D).

Particularly, lysoPC C16:1 and C18:1, which are delta 9 unsaturated fatty acids, were significantly decreased by 43% and 39% in the OB group, and by 63% and 53% in the TM group, respectively, compared to controls. The decrease in lysoPC C20:4 was also significant, showing a 61% reduction in the TM group (Figure 5D).

Surprisingly, though we detected downregulation of short chain acylcarnitine, C18:2 acylcarnitine, a long chain acylcarnitine significantly upregulated 4.4 times in the TM group when compared with the CON group. C3-OH, another short chain acylcarnitine, which is negligible in the CON group, was upregulated 47 times in the OB group but not changed in the TM group when compared with the CON group. C9 acylcarnitine content mildly but significantly decreased by 26% in the OB group but unchanged in the TM group when compared with the CON group (Figure 5E).

PC C32:3 is the only one significantly changed Phosphatidylcholine, it is decreased and increased by 20% and 13% in the OB and the TM group respectively when compared with the CON group (Figure 5F).

## DISCUSSION

This study sets out to investigate the metabolic shifts in cardiac tissues of aging nonhuman primates with spontaneous obesity or TM, focusing on the multi-omics alterations that occur in the context of obesity and TM. Our findings reveal significant changes in the proteomic and metabolomic profiles, highlighting the complex interplay between these conditions and their impact on cardiac metabolism.

The proteomic analysis highlighted distinct alterations in cardiac protein expression associated with TM compared to obesity alone, revealing specific metabolic repercussions of TM on cardiac function. The downregulation of proteins crucial to ion metabolism, including iron regulators AAK1 [19] and transferrin [20], and calcium regulators NUCB1 [21] and CALU [22], suggests a disruption in iron and calcium homeostasis. This metabolic disorder may significantly contribute to the cardiac injury observed in the aging population with TM. Multiple cardiac ECM proteins were found to have significantly decreased, which includes liver-derived proteins such as transferrin, serpin family members, fibrinogen family members, and haptoglobin [23–25]. Notably, a significant proportion of the differentially expressed proteins are primarily synthesized in the liver, including transferrin and various ECM proteins such as serpins and fibrinogen. Since ECM proteins play a critical role in regulating cardiac metabolism through pathways like PI3K-Akt [26–28], our findings suggest a potential disruption in the heart-liver axis via ECM, which may contribute to maintaining cardiac function and metabolism [23].

Our metabolomic and lipidomic analysis underscored the metabolic dysregulation characteristic of TM, particularly affecting fatty acid and amino acid metabolism. The observed reduction in short chain acylcarnitine in both the obesity and TM groups coupled with a significant increase in C18:2 acylcarnitine specifically in the TM group, suggests a disruption in fatty acid oxidation [29], a process essential for cardiac energy production [30, 31]. The contrasting patterns of fatty acid content—increased in the OB group and decreased in the TM group—indicate a shift in energy substrate utilization within cardiac tissue, reflecting the metabolic transition from obesity to TM in the context of aging. This metabolic disorder may contribute to the development of TM and heart failure. The decreased fatty acid content in the TM group is likely a consequence of insulin resistance and heightened fatty acid utilization, further implicating metabolic disturbances in the progression of cardiac dysfunction.

Amino acid metabolism was significantly disrupted in our study, with potentially implications for cardiac function. Alanine, a central player in the Cori cycle that facilitates glucose synthesis from lactate, is crucial for meeting myocardial energy demands during exertion and stress [32]. In the context of coronary artery disease (CAD), an increased myocardial release of alanine correlates with the severity of coronary stenosis, underscoring its role in cardiac metabolism [33]. The observed alterations in alanine levels, along with other amino acids in cardiac tissues, suggest a metabolic shift in myocardial substrate utilization. This shift may represent an adaptive response to metabolic stress imposed by TM. Supporting this, we also found decreased levels of both saturated and unsaturated fatty acids in the TM group, indicative of impaired fatty acid oxidation [4]. Additionally, the reduction in acylcarnitine levels aligns with these findings, pointing to a broader impairment of cardiac metabolic pathways under TM-induced stress. The concurrent disruptions in amino acid and lipid metabolism highlight a comprehensive alteration in the myocardial energy landscape, potentially compromising cardiac function in the setting of TM.

The decrease in lysoPC levels, particularly in the TM group, represents another significant finding of our study. LysoPCs are known to regulate the transport and metabolism of cationic amino acids and play a crucial role in maintaining normal cardiac function [34], including arrhythmia regulation [35, 36] and the induction of arachidonic acid release in cardiac myoblasts [37]. The observed downregulation of arachidonic acid in the TM group’s cardiac tissue further underscores the interconnected nature of these metabolic pathways. Moreover, lysoPC has been identified to inhibit insulin-induced Akt activation in vascular smooth muscle cells [38], suggesting its potential role of decreased lysoPC level in metabolic stress responses and cardiac dysfunction in aging monkeys with TM. These findings emphasize the clinical relevance of lysoPC disruption and highlight the need for further research into its role in cardiac health. Taken together, the concurrent disruption of lysoPC, amino acids, fatty acids, and acylcarnitine in the TM group's cardiac tissue paints a comprehensive picture of metabolic remodeling in the context of TM and aging.

While our study provides valuable insights into the metabolic consequences of obesity and TM on cardiac tissue, it is not without limitations. The small sample size of monkeys with spontaneous TM and obesity, derived from a large population screening, restricts the generalizability of our findings. Additionally, the cross-sectional nature of our study precludes establishing causal relationships between observed metabolic changes and TM pathophysiology. Future studies with larger cohorts and longitudinal designs are needed to validate these observations and elucidate the clinical implications of our multi-omic findings.

In conclusion, our multi-omics approach has revealed the intricate metabolic changes within cardiac tissues associated with obesity and TM. Obesity, particularly aging-related obesity, often closely associated with pre-diabetes stage [39], presents distinct metabolic alterations in cardiac tissue compared to healthy aging controls, including increased fatty acid content but reduced carnitine levels and extracellular matrix (ECM) components. These findings suggest an early disruption in lipid metabolism and ECM integrity, potentially setting the stage for TM development.

**Figure 6:**
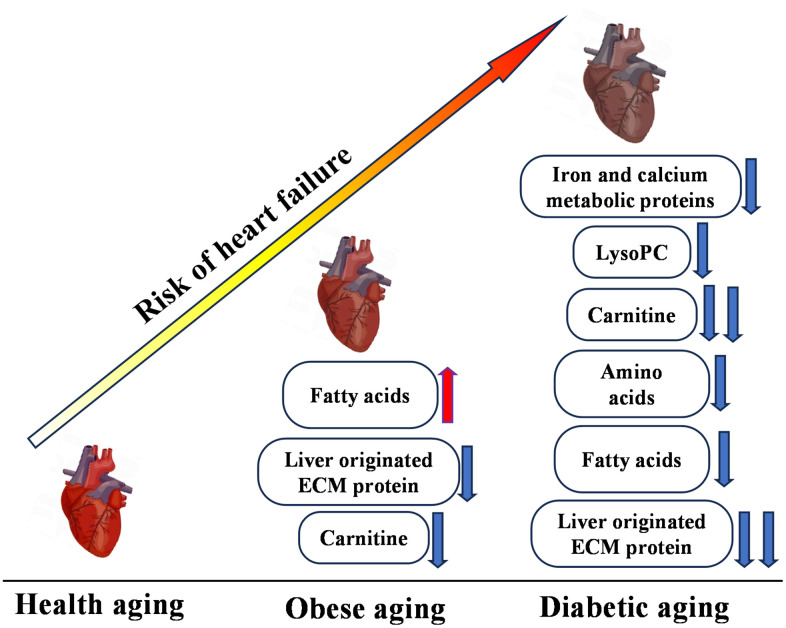
**Multi-omics overview highlighting differences between control (CON), obesity (OB), and type 2 diabetes (TM) groups.** This figure presents a comprehensive summary of the multi-omics changes observed across proteomic, metabolomic, and lipidomic profiles comparing the CON, OB, and TM groups.

The TM group demonstrated more advanced metabolic disturbances, characterized by decreases in iron and calcium-regulatory proteins, lysoPCs, and amino acids, highlighting severe metabolic stress. Notably, the contrasting fatty acid profiles between the obesity and TM groups—an increase in obesity and a decrease in TM—may reflect the progression from insulin resistance to more profound metabolic dysfunction, a hallmark of TM pathophysiology (Figure 6).

These findings contribute to the growing body of evidence that highlights the need for targeted interventions to mitigate the cardiovascular complications of TM. Further research is warranted to understand the mechanisms underlying these metabolic shifts and to develop therapeutic strategies aimed at preserving cardiac function in the context of aging related metabolic disorder.

## MATERIALS AND METHODS

### Generation of spontaneous obese and diabetic rhesus monkeys (*Macaca mulatta*)

Adrenal glands were obtained from rhesus macaques (*Macaca mulatta*) that were raised at the Kunming Institute of Zoology, Chinese Academy of Sciences. The criteria used for screening of spontaneously obese and diabetic rhesus monkeys were as previously described [40]. In brief, body mass index (BMI) ≥ 40 kg/m2 was defined as obesity and plasma levels of fasting blood glucose ≥ 7.0 mmol/L was defined as type 2 diabetes (TM). In total 9 monkeys were included in this study with 3 in each group: healthy male monkeys (CON), obese male monkeys (OB), and TM male monkeys (TM). All the nine monkeys had libitum access to water and food including 21.6% of calories as protein, 5.4% as fat, and 56.6% as carbohydrates. The monkeys were euthanized using 20 mg/kg ketamine, and cardiac muscle was collected (100 mg/animal).

### Metabolomics

Metabolomics analysis was performed using a Q300 Kit (Metabo-Profile, Shanghai, China). Each harvest tissue was stored in an Eppendorf Safelock microcentrifuge tube (Eppendorf, Hamburg, Germany) and mixed with 10 pre-chilled zirconium oxide beads and 20 μL deionized water. Metabolites were extracted using 150 μL methanol containing the internal standard after homogenization of 3 min. Then another 3 mini homogenization was performed and followed by centrifuged at 18,000 × g for 20 min. The supernatants were transferred to 96-well plates and a Biomek 4000 workstation (Biomek 4000; Beckman Coulter Inc., Brea, CA, USA) was used for the following procedures. 20 μL freshly prepared derivative reagent was added into each well. The plate was sealed, and derivatization was conducted at 30° C for 60 min. After derivatization, the samples were evaporated for 2 h and 330 μL ice-cold 50% (v/v) methanol was added to each well to reconstitute the samples. The plate was stored at − 20° C for 20 min and the samples were centrifuged at 4000 × g and 4° C for 30 min. Then, 135 μL supernatant was transferred to a new 96-well plate with 15 μL internal standards in each well. Serial dilutions of derivatized stock standards were added to the wells on the left side and the plate was sealed for liquid chromatography-mass spectrometry (LC–MS) analysis. All metabolite targets were quantitated using an ultraperformance liquid chromatography coupled with tandem mass spectrometry (UPLC-MS/MS) system (ACQUITY UPLC-Xevo TQ-S; Waters Corp., Milford, MA, USA) by Metabo-Profile Biotechnology (Shanghai) Co. Ltd.

Raw data files generated by UPLC-MS/MS were processed using the QuanMET software (v2.0; Metabo-Profile, Shanghai, China) to obtain peak integration, calibration and quantitation for each metabolite.

### Lipidomic

We performed the lipidomics analysis with primary targeted metabolic profiling platform BAP Ultra (Metabo-Profile, Shanghai, China). Harvested samples were stored in an Eppendorf Safelock microcentrifuge tube (Eppendorf, Hamburg, Germany) and centrifuged at 2000 rcf for 10 min. After that, the supernatant was collected and transferred to a 96-well plate. All samples were qualified after quality control. Then the lipidomics profile was detected using Ultra Performance Liquid Chromatography Tandem Mass Spectrometer (UPLC-MS/MS) with normalization based on mass following the manufacturer's instructions by Metabo-Profile Inc. (Shanghai, China).

Raw data were processed by an automation assay workflow using LipidMET software streamlines and lipids in the samples were identified and quantitated for further analysis.

### Proteome

Protein was extracted using SDT method (4% sodium dodecyl sulfate [SDS], 100 mmol/L Tris/HCl, 0.1 mol/L DTT, pH 7.6). After trypsin digested with FASP (Filter aided proteome preparation), the peptides were desalted and reconstituted with 40 μL of 0.1% formic acid solution. Then the peptides were labeled using the TMTsixplex™ Isobaric Label Reagent Set (#90111, Thermo Fisher Scientific, USA). TMT-labeled peptides were fractionated using high pH Reversed-Phase Peptide Fractionation Kit (#84868, Thermo Fisher Scientific). After desalting and vacuum drying, the peptide was reconstituted using 12 μL of 0.1% fatty acid (FA) and concentration was based on ultraviolet (UV) absorbance at 280 nm. The TMT-labeled peptide set was blended using AKTA Purifier 100 (GE, Uppsala, Sweden) and subjected to chromatographic column for fractionation. The prepared samples were then used for liquid chromatography with tandem mass spectrometry (LC-MS/MS) and mass spectrometric analysis using a Q-Exactive mass spectrometer.

Raw peptides were identified and quantitated using Maxquant software (https://www.maxquant.org/, version 1.5.1.0).

### Western blots

Proteins were extracted from left ventricle samples using RIPA buffer supplemented with protease inhibitors. Protein concentration was determined by BCA assay. Samples (20–50 μg) were denatured in Laemmli buffer, resolved by 10% SDS-PAGE, and transferred to a PVDF membrane. The membrane was blocked with 5% non-fat milk in TBST and incubated overnight at 4° C with primary antibodies from Proteintech (USA): rabbit anti-transferrin (1:1000), rabbit anti-ORM1 (1:1000), rabbit anti-C3 (1:1000), rabbit anti-NUCB1 (1:1000), rabbit anti-SERPINA1 (1:1000), or mouse anti-GAPDH (1:5000). After washing, the membrane was incubated with HRP-conjugated secondary antibody (1:5000) for 1 hour at room temperature. Bands were visualized using ECL and imaged with a ChemiDoc system.

### Data availability

The datasets generated and analyzed during the current study are not publicly available due to restrictions, such as containing information that could compromise the privacy of research participants or proprietary considerations. However, these datasets are available from the corresponding author upon reasonable requests and for researchers who meet the criteria for access to confidential data. The mass spectrometry proteomics data have been deposited in Dryad with the accession code DOI: 10.5061/dryad.c2fqz61ks. Metabolomics and lipidomics data are also deposited in Dryad under the same identifier, facilitating integrated access to related datasets.

### Data analysis

All data analyses were performed in language R. The significance was assessed using Student’s t-test with a p-value of < 0.05. The heatmap, boxplot and volcano were conducted using package “pheatmap” and “ggplot” respectively.

## Supplementary Material

Supplementary Figures

Supplementary Table 1

Supplementary Tables 2 and 3

Supplementary Table 4
