# Supplementary material for: Multi-omics analysis reveals metabolic disruptions in cardiac tissues of aging nonhuman primates with spontaneous type 2 diabetes
Source: Aging (Albany NY). 2025 Jun 2;17(6):1386–404. doi: 10.18632/aging.206261 (PMC12245199; doi:10.18632/aging.206261)
Supplement: Supplementary Figures [file aging-17-206261-s001.pdf]

SUPPLEMENTARY FIGURES

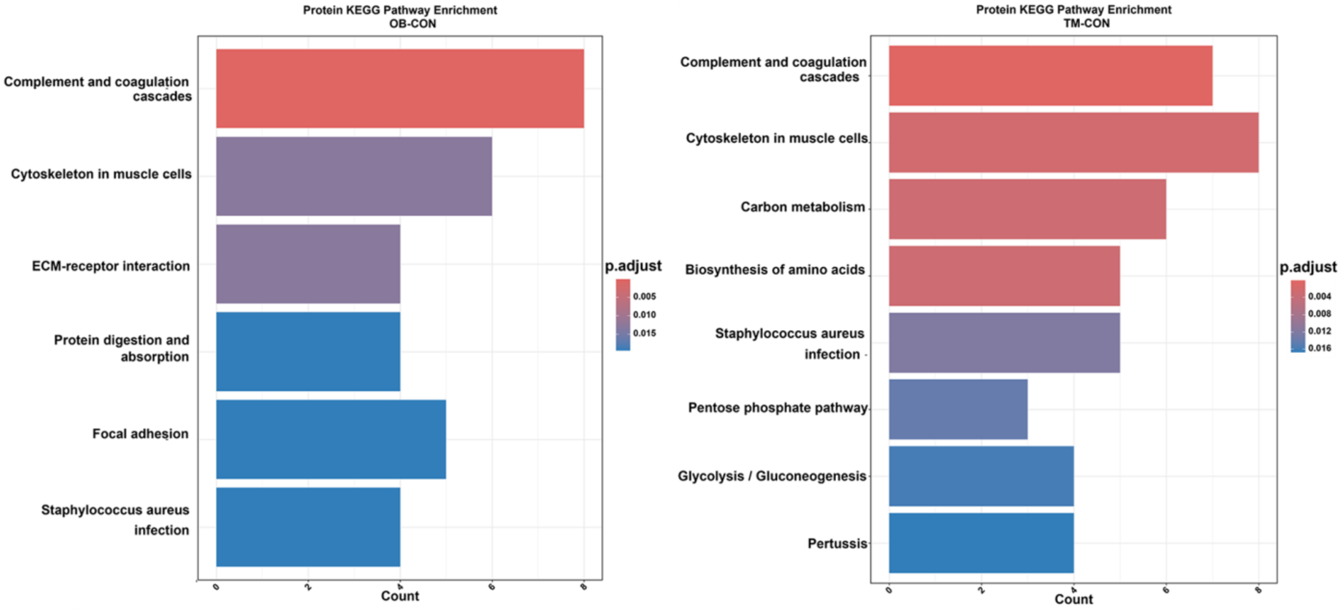

Supplementary Figure 1. Comparative KEGG analysis of proteomics data. This figure displays the pairwise KEGG (Kyoto Encyclopedia of Genes and Genomes) analysis from the proteomics study. The left panel illustrates the KEGG analysis comparing the OB group to the CON group, while the right panel presents the comparison between the TM group and the CON group.

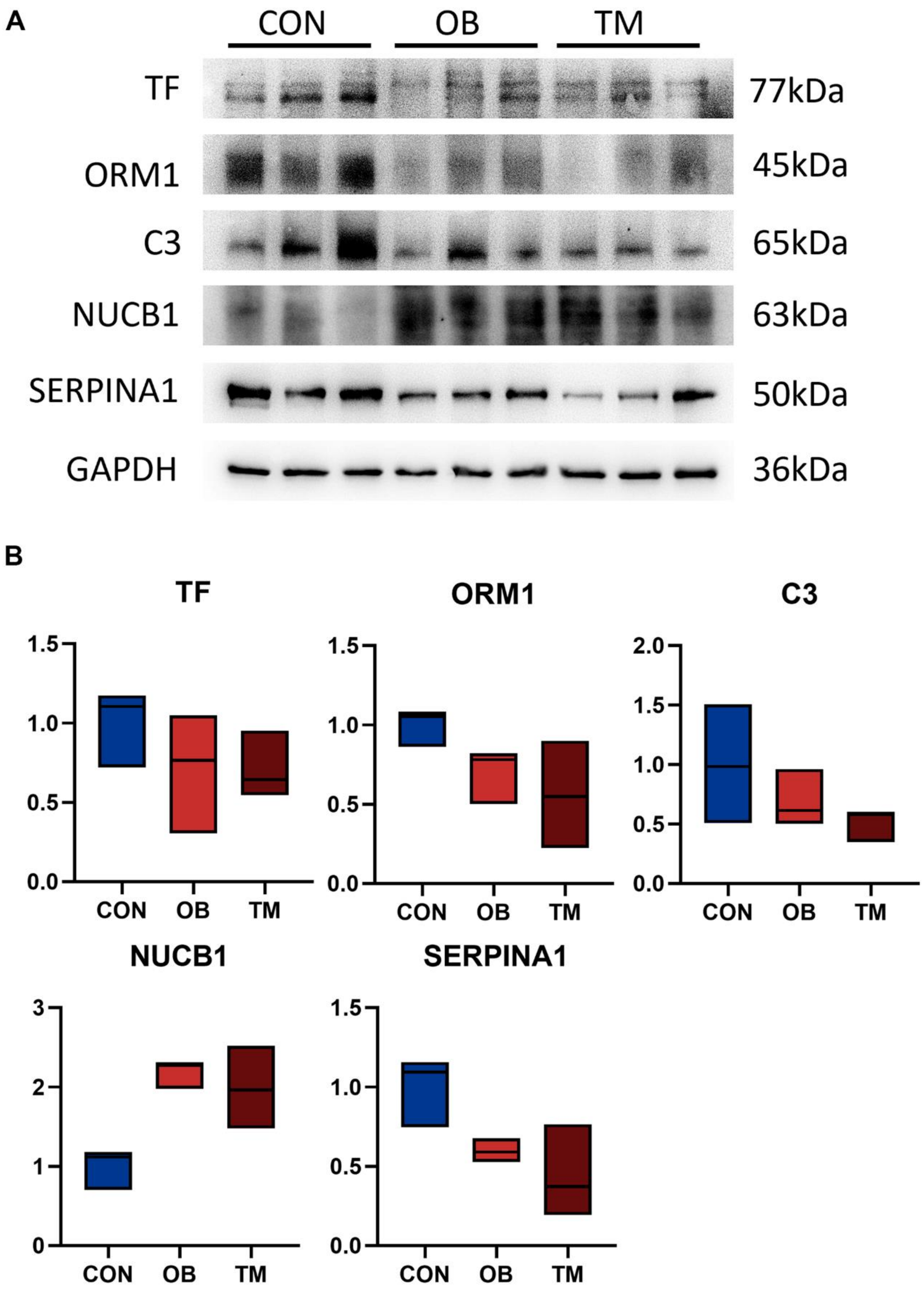

Supplementary Figure 2. Western blot validation of key proteins in aging monkey cardiac tissue. (**A**) Representative Western blot images for transcription factor (TF), orosomucoid 1 (ORM1), complement component 3 (C3), nucleobindin 1 (NUCB1), and serpin family A member 1 (SERPINA1) in left ventricular samples from aging control (CON), obesity (OB), and triglyceride metabolism (TM) groups. (**B**) Quantification of protein expression levels from the blots in (**A**), normalized to a loading control, confirming trends observed in proteomic analysis (n=3).

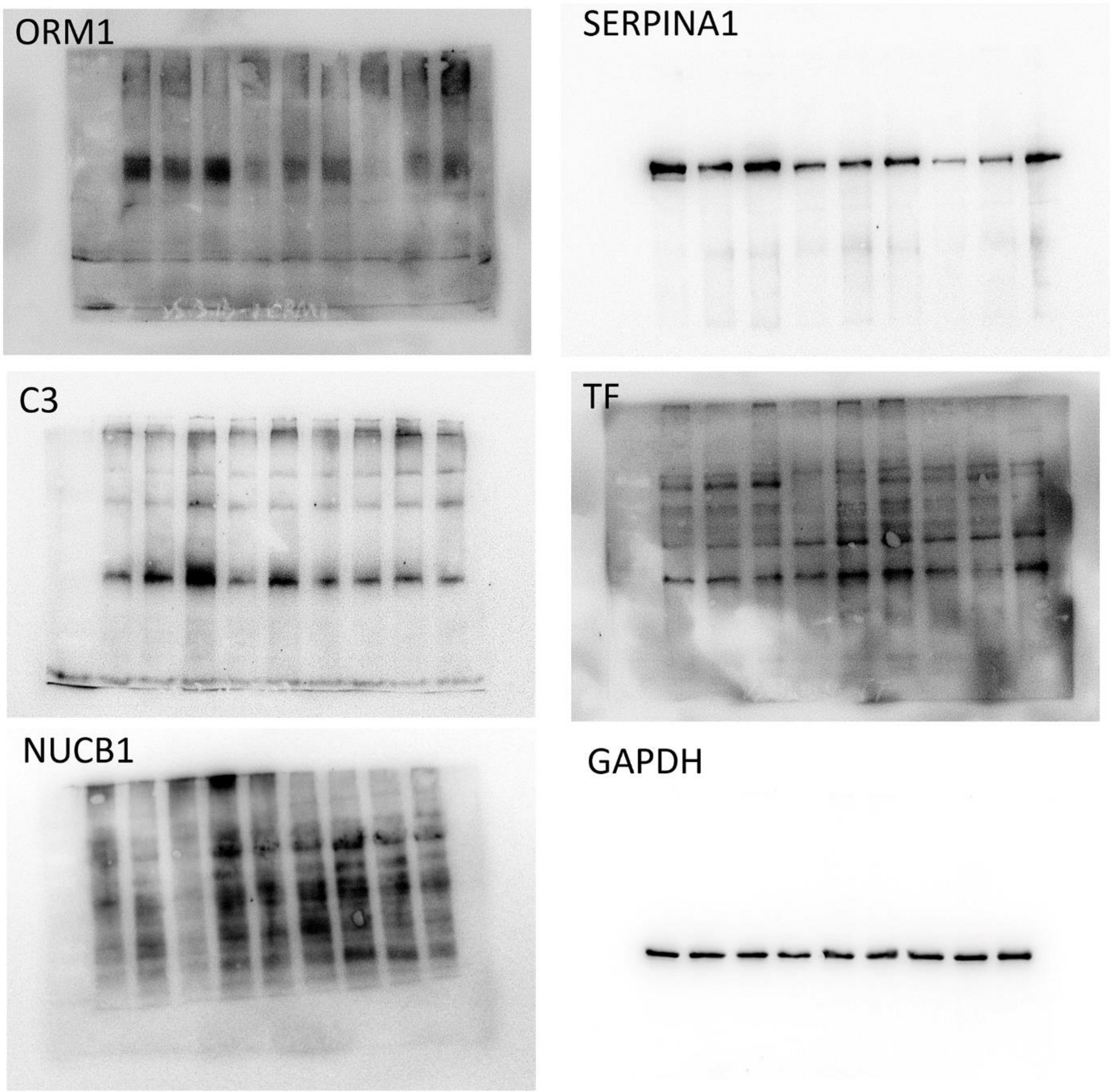

Supplementary Figure 3. Uncropped western blot images for validation study. Full-length, uncropped Western blot membranes corresponding to the proteins analyzed in Supplementary Figure 2—transcription factor (TF), orosomucoid 1 (ORM1), complement component 3 (C3), nucleobindin 1 (NUCB1), and serpin family A member 1 (SERPINA1)—from left ventricular tissue of aging control (CON), obesity (OB), and triglyceride metabolism (TM) groups. Molecular weight markers are indicated.
