# Supplementary material for: Multi-omics analysis reveals metabolic disruptions in cardiac tissues of aging nonhuman primates with spontaneous type 2 diabetes
Source: Aging (Albany NY). 2025 Jun 2;17(6):1386–404. doi: 10.18632/aging.206261 (PMC12245199; doi:10.18632/aging.206261)
Supplement: Supplementary Tables 2 and 3 [file aging-17-206261-s003.pdf]

Supplementary Table 2 Significantly altered metabolites in the OB and TM groups versus the CON group.

Class	Name	p-value
acylcarnitines	C18:2	0.0115198
acylcarnitines	C3-OH	0.000406042
acylcarnitines	C9	0.00941521
glycerophospholipids	lysoPC a C16:0	0.0023923
glycerophospholipids	lysoPC a C16:1	0.00293587
glycerophospholipids	lysoPC a C17:0	0.0288291
glycerophospholipids	lysoPC a C18:0	0.0134699
glycerophospholipids	lysoPC a C18:1	0.0326835
glycerophospholipids	lysoPC a C18:2	0.000341347
glycerophospholipids	lysoPC a C20:4	0.0493653
glycerophospholipids	lysoPC a C28:1	0.0415455
glycerophospholipids	PC aa C32:3	0.000194646
glycerophospholipids	PC ae C40:5	0.0295766

Supplementary Table 3. Significantly altered lipid species in the OB and TM groups versus the CON group.

	FBG(mmol/L)	BW(Kg)	BMI(Kg/m^2)	ST(mmol/L)	SC(mmol/L)	LDL-c(mmol/L)	HDL-c(mmol/L)
	3.8	5.1	27.4	0.54	2.91	1.58	0.98
CON	3.5	5.2	28.6	0.65	3.85	2.25	1.17
	4.1	4.8	25.5	0.57	2.06	1.12	0.65
	5	16.1	50.5	2.04	4.52	1.71	1.33
OB	4.9	12.6	46.4	1.92	3.85	2.31	1.42
	4.9	14.2	44.1	1.88	3.02	1.42	1.25
	8.2	7.1	26.2	0.85	3.05	1.48	1.05
TM	7.7	7.3	27.7	1.18	3.65	1.31	1.28
	8.9	7.7	25.5	0.96	2.27	1.81	0.86
